# Hybrid regularizers-based adaptive anisotropic diffusion for image denoising

**DOI:** 10.1186/s40064-016-1999-6

**Published:** 2016-04-02

**Authors:** Kui Liu, Jieqing Tan, Liefu Ai

**Affiliations:** School of Computer and Information, Hefei University of Technology, Tunxi Road, 23009 Hefei, China; The Key Lab of Intelligent Perception and Computing of Anhui Province, Jixian Road, 246011 Anqing, China

**Keywords:** Image denoising, Total variation, Fourth-order filter, Split Bregman method, Relaxation method

## Abstract

To eliminate the staircasing effect for total variation filter and synchronously avoid the edges blurring for fourth-order PDE filter, a hybrid regularizers-based adaptive anisotropic diffusion is proposed for image denoising. In the proposed model, the $H^{-1}$-norm is considered as the fidelity term and the regularization term is composed of a total variation regularization and a fourth-order filter. The two filters can be adaptively selected according to the diffusion function. When the pixels locate at the edges, the total variation filter is selected to filter the image, which can preserve the edges. When the pixels belong to the flat regions, the fourth-order filter is adopted to smooth the image, which can eliminate the staircase artifacts. In addition, the split Bregman and relaxation approach are employed in our numerical algorithm to speed up the computation. Experimental results demonstrate that our proposed model outperforms the state-of-the-art models cited in the paper in both the qualitative and quantitative evaluations.

## Introduction

With the popularity of image sensor, digital images play a key role in people’s daily life. Unfortunately, images are ineluctably contaminated by noise during acquisition, transmission, and storage. Therefore, image denoising is still an open and complex problem in image processing and computer vision (Chatterjee and Milanfar 2010). Image denoising aims to recovering the original image *u* from the observed noisy image $u_0$, where $u_0=u+n$, and *n* is the zero-mean Gaussian white noise with standard deviation $\sigma$.

During the past three decades, lots of approaches for removing noise have been developed from linear models to nonlinear models. Linear models perform well in the smooth area. However, they don’t preserve edges and corners. To overcome the disadvantages of the linear denoising models, nonlinear denoising models have been developed which have a good balance between noise removal and edge-preserving. Nonlinear models based on variation (Rudin et al. 1992) and partial differential equation (PDE) (Perona and Malik 1990) have been widely used for image denoising. The best known variational denoising model is the total variation (TV) model proposed by Rudin et al. (1992), which minimizes the following equation,

1 $$\min \limits _{u}\left\{ \int _\Omega \left( |\nabla u|+\frac{\lambda }{2}(u-u_0)^2\right) d\Omega \right\}$$

where $\Omega \subseteq R^2$ is a bounded open domain with Lipschitz boundary, $\nabla$ denotes the gradient operator, $|\nabla u|$ is the TV regularization term, $(u-u_0)^2$ is the fidelity term, $\lambda >0$ is the regularization parameter, which measures the trade off between the regularization term and the fidelity term.

The classical TV model is efficient for removing noise and preserving the edges. However, it possesses some undesirable properties in the recovered image under some circumstances, such as the staircasing effect. To overcome the deficiency of the original TV model (Strong 1997), developed the adaptive TV regularization based variational model as,

2 $$\min \limits _u\int _\Omega \left( g(x)|\nabla u|+\frac{\lambda }{2}(u-u_0)^2\right) d\Omega$$

where *g*(*x*) is an adaptive edge-stopping function, which is defined in Strong (1997) as follow,

3 $$g(x)=\frac{1}{1+{\mathcal {K}}|\nabla G_\rho (x)*u_0|^2},$$

where ${\mathcal {K}}>0$ is a threshold parameter for balancing the noise removal and edge preservation, and $G_\rho (x)$ is the Gaussian filter with standard deviation $\rho$. Seen from (3), *g*(*x*) is smaller near the edges and larger away from the boundaries, so the model of (2) has the capability of preserving the edges while removing noise because the diffusion is stopped across edges.

In addition, Nikolova replaced the $\ell ^2$-norm with the $\ell ^1$-norm in the fidelity term of TV model in Nikolova (2002). Osher et al. (2005) proposed an iterative regularization method of TV model. Chen et al. (2010) presented an adaptive total variation method based on the difference curvature. Wang et al. (2011) put forward a modified TV model.

Numerical experiments demonstrate that the models mentioned above have good performance in the terms of the trade-off between removing noise and preserving the edges. Unfortunately, the staircasing effects appear in the recovered image owing to using the TV-norm as the regularization term. To overcome this shortcoming, high-order PDE filters have been proposed and applied for image denoising successfully (Lysaker et al. 2003; Liu et al. 2011). One of the most classical fourth-order PDEs (LLT) is introduced by Lysaker et al. (2003)

4 $$\int _\Omega \left( |\nabla ^2 u|+\frac{\lambda }{2}(u-u_0)^2\right) d\Omega$$

where $\nabla ^2$ denotes the Laplacian operator. However, a major challenge is that higher-order PDEs blur the edges during image denoising.

To make use of the advantages of both TV filter and high-order PDE filters, some hybrid regularization models are recently proposed, which combined the second-order partial differential equations and the fourth-order partial differential equations (Oh et al. 2013). Li et al. (2007) proposed the adaptive image denoising model based on hybrid regularizers combining the advantages of TV model and LLT model as follows,

5 $$\min \limits _u\int _\Omega \left( (1-g)|\nabla u|+g|\nabla ^2 u|+\frac{\lambda }{2}(u-u_0)^2\right) d\Omega$$

where *g*(*x*) also denotes the edge-stopping function defined as in (3). The results of experiments indicate that the model of (5) performs better than the pure second-order or hight-order models.

In recent years, efficient computational algorithms for solving the denoising models have emerged in large numbers, for instance, fixed point iteration, gradient descent methods, primal-dual methods, relaxation methods, Bregman iteration and split Bregman method, and so on. These methods are efficient for image denoising while preserving the edges.

Inspired by Li et al. (2007) and Liu (2015), we propose a novel adaptive anisotropic diffusion model, incorporating the advantages of the total variation filter and the fourth-order filter, and develop an efficient computational algorithm. The main contributions of our paper can be generalized as follows. First of all, the hybrid regularization term of the novel model is composed of total variation regularization and a fourth-order filter. The fidelity term uses the $H^{-1}$-norm as opposed to the more commonly used $\ell ^1$-norm or $\ell ^2$-norm. The two above-mentioned filters can be adaptively selected according to the diffusion function. When the pixels locate at the edges, the total variation filter is selected to filter the image, which can preserve the edges. When the pixels belong to the flat regions, the fourth-order filter is adopted to smooth the image, which can eliminate the staircase artifacts. Another main contribution is that the split Bregman and relaxation approach are successively employed in our numerical algorithm to speed up the computation. Experimental results demonstrate that our proposed model achieves higher quality in both the qualitative and quantitative aspects than that of the state-of-the-art models cited in the paper.

The remainder of this paper is organized as follows. In “Preliminaries” section, we give some definitions. In “The new model and algorithms” section, we give the proposed model and numerical implementation in detail. The experimental results are given in “Experiments” section. Finally, this paper is concluded in the fifth section.

## Preliminaries

In this section, we give a brief overview of some necessary notations and definitions for the proposed model, which will be used in the subsequent sections.

### **Definition 1**

(Chen and Wunderli 2002). Let $\Omega$ be an open bounded subset of ${\mathbb {R}}^n(n\ge 2)$ with Lipschitz boundary. Given a function $u\in L^1(\Omega )$. Then the total variation of *u* in $\Omega$ is defined as,

6 $$\int _\Omega |\nabla u|:=\sup \left\{ \int _\Omega u div\phi d\Omega |\phi \in C^1_c(\Omega ,{\mathbb {R}}^n),\Vert \phi \Vert _{L^\infty (\Omega )}\le 1\right\},$$

where *div* is the divergence operator, $C_c^1(\Omega ,{\mathbb {R}}^n)$ is the subset of continuously differentiable vector functions of compact support contained in $\Omega$, and $L^\infty (\Omega )$ is the essential supremum norm.

### *Remark 1*

Let the Sobolev space be $W ^{1,1}(\Omega ):=\{u\in L^1(\Omega )|\nabla u\in L^1(\Omega )\}$. If $\Vert u\Vert _{BV^2(\Omega )}=\int _\Omega |\nabla u|+\Vert u\Vert _{ W ^{1,1}(\Omega )}$, the space $BV^2(\Omega )$ is a Banach space.

### **Definition 2**

(Liu et al. 2007). Let $\Omega$ be an open bounded subset of ${\mathbb {R}}^n(n\ge 2)$ with Lipschitz boundary. Given a function $u\in L^1(\Omega )$. Then the $BV^2$ seminorm of *u* is defined as,

7 $$\int _\Omega |\nabla ^2 u|:=\sup \left\{ \int _\Omega <\nabla u, div(\varphi )>_{{\mathbb {R}}^n}|\varphi \in C^2_c(\Omega ,{\mathbb {R}}^{n\times n} ),\Vert \varphi \Vert _{L^\infty (\Omega )}\le 1\right\} ,$$

where

8 $$div(\varphi ):=(div(\varphi _1),div(\varphi _2),\cdots ,div(\varphi _n)),$$

with $\forall i,\varphi _i=\{\varphi ^1_i,\cdots ,\varphi ^n_i)$ and $div(\varphi _i)=\sum _{j=1}^{n}\frac{\partial \varphi ^j_i}{\partial x_j}$, and $\Vert \varphi \Vert =\sqrt{\sum _{i,j=1}^{n}(\varphi _i^j)^2}$.

### **Definition 3**

(Liu 2015). Let $\Omega$ be an open subset of ${\mathbb {R}}^n(n\ge 2)$ with Lipschitz boundary. Given a function $u\in L^1(\Omega )$, and let $\alpha (x)\ge 0$ be a continuous real function. Then the $\alpha -$total Variation of *u* in $\Omega$ is defined by,

9 $$\int _\Omega \alpha |\nabla u|:=\sup \left\{ \int _\Omega udiv\phi d\Omega |\phi \in C^1_c(\Omega ,{\mathbb {R}}^n),\Vert \phi _i\Vert _{L^\infty (\Omega )}\le \alpha ,1\le i\le n\right\} ,$$

where the vector valued function $\phi =(\phi _1,\phi _2,\ldots ,\phi _n)$. Moreover, the $\alpha -BV$ seminorm is characterized by $\Vert u\Vert _{\alpha -BV}=\int _\Omega \alpha |\nabla u|+\Vert u\Vert _{L^1(\Omega )}$.

### **Definition 4**

(Liu 2015). Let $\Omega$ be an open subset of ${\mathbb {R}}^n(n\ge 2)$ with Lipschitz boundary. Given a function $u\in L^1(\Omega )$, and let $\beta (x)\ge 0$ be a continuous real function. Then the weighted $BV^2$ seminorm of *u* in $\Omega$ is defined as,

10 $$\int _\Omega \beta |\nabla ^2 u|:=\sup \left\{ \int _\Omega <\nabla u, div(\varphi )>_{{\mathbb {R}}^n}|\varphi \in C^2_c(\Omega ,{\mathbb {R}}^{n\times n} ),\Vert \varphi \Vert _{L^\infty (\Omega )}\le \beta \right\} ,$$

and $$\Vert u\Vert _{\beta -BV^2(\Omega )}=\int _\Omega \beta |\nabla ^2 u|+\Vert u\Vert _{ W ^{1,1}(\Omega )}$$.

### **Definition 5**

(Jia et al. 2011). For $\lambda >0$ and $c\in {\mathbb {R}}$, the soft thresholding operator $cut(c,\frac{1}{\lambda })$ is defined as,

11 $$\begin{aligned} cut\left( c,\frac{1}{\lambda }\right) = \left\{ \begin{array}{ll} \frac{1}{\lambda }& \quad if\quad c>\frac{1}{\lambda },\\ c&\quad if\quad -1/\lambda \le c\le \frac{1}{\lambda }, \\ -\frac{1}{\lambda }&\quad if \quad c<-\frac{1}{\lambda }. \end{array}\right. \end{aligned}$$

## The new model and algorithms

### The proposed model

**Fig. 1:**
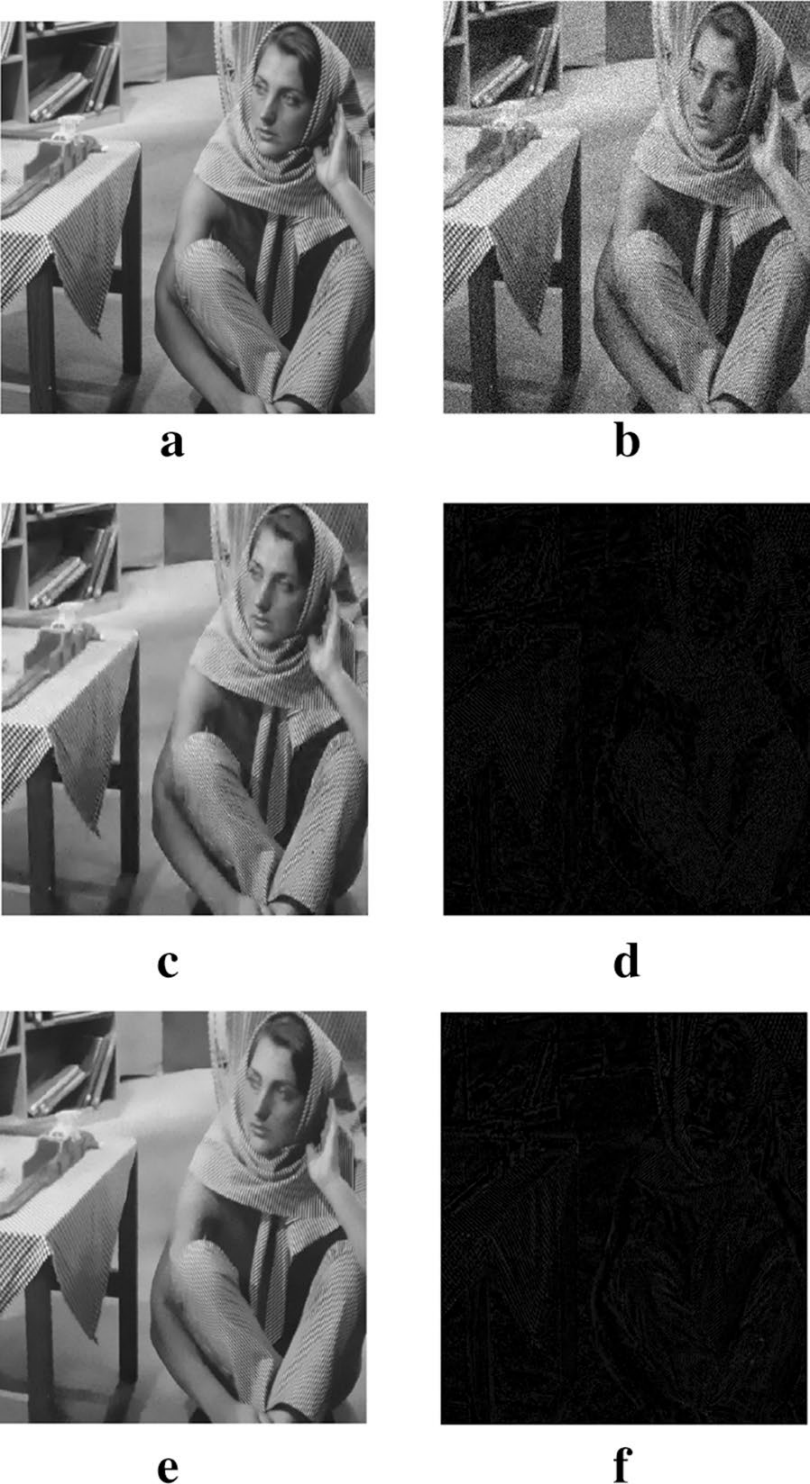
Results of image denoising by our model and model from Li et al. (2007). **a** Original image, **b** noisy image with $\sigma =25$, **c** result by our model, **d** noise, detecting by our model, **e** result by model from Li et al. (2007), **f** noise, detecting by model from Li et al. (2007)

Meyer analyzed that there exists no oscillation function in the space $L^2(\Omega )$ and a weaker $H^{-1}$-norm is appropriate to represent textured or oscillatory patterns (Meyer 2001), so that we replace $\ell ^2$-norm of the fidelity term $(u_0-u)$ with $H^{-1}$-norm. Therefore, a novel adaptive image denoising model is proposed,

12 $$\min \limits _u\left\{ E(u)=\int _\Omega ((1-g(x))|\nabla u|+g(x)|\nabla ^2 u|)d\Omega +\frac{\lambda }{2}\Vert u_0-u\Vert ^2_{H^{-1}}\right\} ,$$

where *u* and $u_0$ are the recovered image and the noisy image, respectively. Seen from Eq. (12), the $H^{-1}$-norm is considered as the fidelity term and the regularization term is composed of a total variation regularization and a fourth-order filter in the proposed model. $\Vert u_0-u\Vert ^2_{H^{-1}}=\int _\Omega |\nabla (\Delta ^{-1}(u_0-u))|^2d\Omega$, and $\Delta ^{-1}$ is the inverse Laplace operator. The diffusivity function *g*(*x*) is defined as,

13 $$g(x)=\exp (-{\mathcal {K}}|\nabla G_\rho *u_0|^2),$$

where Gaussian filter $G_\rho (x)$ pre-smooths the noisy image. The larger standard deviation $\sigma$ of the noise is, the larger standard deviation $\rho$ of Gaussian filter is. We set $\rho =C\sigma$, where *C* lies between 0 to 1. When $g(x)\rightarrow 0$, it means that the pixels locate at the edges. Then total variation filter is selected to filter the image, which can preserve the edges. When $g(x)\rightarrow 1$, it means that the pixels belong to the flat regions. Then the fourth-order filter is adopted to smooth the image, which can eliminate the staircase artifacts. We replace *g*(*x*) with *g* in the next part of this article. Figure 1 shows the results of image denoising by our proposed model and model from Li et al. (2007), which demonstrates that the model whose fidelity term uses the $H^{-1}$-norm yields better results in image denoising since $H^{-1}$-norm is appropriate to represent textured or oscillatory patterns.

### The numerical algorithm for the proposed model

We apply a split Bregman method (Cai et al. 2009) to solve Eq. (12). The idea of split Bregman method is to use splitting operator and Bregman iteration to solve various inverse problems (Goldstein and Osher 2009).

We turn Eq. (12) into the following constrained minimization problem by introducing an auxiliary variable *z*,

14 $$\min \limits _u\left\{ \int _\Omega \left( (1-g)|\nabla u|+g|\nabla ^2 u|+\frac{\lambda }{2}|\nabla (\Delta ^{-1}(u_0-z))|^2\right) d\Omega \right\} ,s.t. z=u.$$

The method of solving the constrained minimization problem is that it may be transformed into the unconstrained minimization problem, so the constrained problem (14) can be turned into the following unconstrained problem by introducing an auxiliary variable *b*,

15 $$\min \limits _{u,z,b}\left\{ \int _\Omega \left( (1-g)|\nabla u|+g|\nabla ^2 u|+\frac{\lambda }{2}|\nabla (\Delta ^{-1}(u_0-z))|^2\right) d\Omega +\frac{\mu }{2}\Vert u-z+b\Vert {^2_2}\right\} ,$$

By taking advantage of split Bregman method, Eq. (15) can be solved iteratively according to the following equations,

16 $$\begin{aligned} \left\{ \begin{array}{rcl} u^{k+1}&=&\min \nolimits _u \left\{ \int _\Omega ((1-g)|\nabla u|+g|\nabla ^2 u|) d\Omega +\frac{\mu }{2}\Vert u-z^k+b^k\Vert {^2_2}\right\} , \\ z^{k+1}&{}=&{}\min \nolimits _z\left\{ \frac{\lambda }{2}\int _\Omega |\nabla ( \Delta ^{-1}(u_0-z))|^2d\Omega +\frac{\mu }{2}\Vert u^{k+1}-z+b^k\Vert {^2_2}\right\} ,\\ b^{k+1}&=&b^k+u^{k+1}-z^{k+1}, \end{array}\right. \end{aligned}$$

where *k* is the number of iterations.

#### Solve the first subproblem in Eq. (16)

At present, the Euler–Lagrange equation method is usually used to solve the problem similarity to the first subproblem in Eq. (16). However, it works slowly. To accelerate the computation speed, the split Bregman algorithm and relaxation algorithm are adopted to solve the first subproblem in Eq. (16).

First, we define $|\nabla u|=\Vert \nabla _x u\Vert _1+\Vert \nabla _y u\Vert _1$, and $|\nabla ^2 u|=\Vert \Delta _x u\Vert _1+\Vert \Delta _y u\Vert _1$, and then the first subproblem in Eq. (16) can be rewritten as follows,

17 $$u^{k+1}=\min \limits _u\left\{ (1-g)(\Vert \nabla _x u\Vert _1+\Vert \nabla _y u\Vert _1)+g(\Vert \Delta _x u\Vert _1+\Vert \Delta _y u\Vert _1)+\frac{\mu }{2}\Vert u-z^k+b^k\Vert {^2_2}\right\} ,$$

where $\nabla _x$, $\nabla _y$, $\Delta _x$ and $\Delta _y$ are the first-order difference operators and the second-order difference operators, respectively. All the difference operators are approximated using following formulas:

18 $$\begin{aligned} \nabla _x u_{i,j}&=\left\{ \begin{array}{ll} 0&\quad if\quad i=1, \\ u_{i,j}-u_{i-1,j}&{}\quad if\quad 1<i\le M, \end{array}\right. \end{aligned}$$

19 $$\begin{aligned} \nabla _y u_{i,j}&=\left\{ \begin{array}{ll} 0& \quad if\quad j=1,\\ u_{i,j}-u_{i,j-1}& \quad if\quad 1<j\le N, \end{array}\right. \end{aligned}$$

20 $$\begin{aligned} \Delta _x u_{i,j}&=\left\{ \begin{array}{ll} u_{1,j}-u_{2,j}&{}\quad if\quad i=1, \\ 2u_{i,j}-u_{i-1,j}-u_{i+1,j}& \quad if\quad 1<i< M, \\ u_{M-1,j}-u_{M,j}&\quad if\quad i=M \end{array}\right. \end{aligned}$$

21 $$\begin{aligned} \Delta _y u_{i,j}&=\left\{ \begin{array}{ll} u_{i,1}-u_{i,2}&\quad if\quad j=1,\\ 2u_{i,j}-u_{i,j-1}-u_{i,j+1} &\quad if\quad 1<j<N, \\ u_{i,N-1}-u_{i,N}&\quad if\quad j=N. \end{array}\right. \end{aligned}$$

where $M\times N$ represents the image size.

Second, we introduce four auxiliary variables $\upsilon _x, \upsilon _y, \omega _x,$ and $\omega _y$, and then Eq. (17) can be transformed into the following constrained optimization problem,

22 $$u^{k+1}=\min \limits _u\left\{ (1-g)(\Vert \upsilon _x \Vert _1+\Vert \upsilon _y\Vert _1)+ g(\Vert \omega _x\Vert _1+\Vert \omega _y\Vert _1)+ \frac{\mu }{2}\Vert u-z^k+b^k\Vert {^2_2}\right\} ,$$

with $$\upsilon _x=\nabla _x u, \upsilon _y=\nabla _y u, \omega _x=\Delta _x u,$$ and $$\omega _y=\Delta _y u$$.

The above constrained problem (22) are turned into the unconstrained minimization problem,

23 $$\begin{aligned} u^{k+1}=&\min \limits _{\upsilon _x,\upsilon _y,\omega _x,\omega _y,u}\left\{ (1-g)(\Vert \upsilon _x \Vert _1+\Vert \upsilon _y\Vert _1)+ g(\Vert \omega _x\Vert _1+\Vert \omega _y\Vert _1)+ \frac{\mu }{2}\Vert u-z^k+b^k\Vert ^2_2\right. \nonumber \\&\quad +\frac{\alpha }{2}\Vert \upsilon _x-\nabla _x u-f^k_x\Vert ^2_2 + \frac{\alpha }{2}\Vert \upsilon _y-\nabla _y u-f^k_y\Vert ^2_2\nonumber \\&\left. \quad +\frac{\beta }{2}\Vert \omega _x-\Delta _x u-c^k_x\Vert ^2_2+ \frac{\beta }{2}\Vert \omega _y-\Delta _y u-c^k_y\Vert ^2_2\right\} , \end{aligned}$$

where the parameters $\alpha >0$ and $\beta >0$. Let $F(\upsilon _x, \upsilon _y,\nabla _x u, \nabla _y u)= \frac{\alpha }{2}\Vert \upsilon _x-\nabla _x u-f^k_x\Vert ^2_2 + \frac{\alpha }{2}\Vert \upsilon _y-\nabla _y u-f^k_y\Vert ^2_2$ and $E(\omega _x, \omega _y,\Delta _x u,\Delta _y u)=\frac{\beta }{2}\Vert \omega _x-\Delta _x u-c^k_x\Vert ^2_2+ \frac{\beta }{2}\Vert \omega _y-\Delta _y u-c^k_y\Vert ^2_2$, and apply the split Bregman method, Eq. (23) can be solved by following equations,

24 $$\begin{aligned} \left\{ \begin{array}{rcl} u^{k+1}&{}=&\min \nolimits _u\left\{ \frac{\mu }{2}\Vert u-z^k+b^k\Vert ^2_2+ F(\upsilon _x^k, \upsilon _y^k,\nabla _x u, \nabla _y u)+E(\omega _x^k, \omega _y^k,\Delta _x u,\Delta _y u)\right\} ,\\ (\upsilon _x^{k+1},\upsilon _y^{k+1})&=&\min \limits _{\upsilon _x,\upsilon _y}\{(1-g)(\Vert \upsilon _x \Vert _1+\Vert \upsilon _y\Vert _1)+F(\upsilon _x,\upsilon _y,\nabla _x u^{k+1}, \nabla _y u^{k+1})\},\\ (\omega _x^{k+1},\omega _y^{k+1})&=&\min \nolimits _{\omega _x,\omega _y}\{g(\Vert \omega _x\Vert _1+\Vert \omega _y\Vert _1)+ E(\omega _x, \omega _y,\Delta _x u^{k+1},\Delta _y u^{k+1})\}, \end{array}\right. \end{aligned}$$

with the update equations,

25 $$\left\{ \begin{array}{lll} f_x^{k+1}&=&f^k_x-(\upsilon ^{k+1}_x-\nabla u^{k+1}_x),\quad f_y^{k+1}=f^k_y-(\upsilon ^{k+1}_y-\nabla u^{k+1}_y), \\ c_x^{k+1}&=&c^k_x-(\omega ^{k+1}_x-\Delta u^{k+1}_x),\quad c_y^{k+1}=c^k_y-(\omega ^{k+1}_y-\Delta u^{k+1}_y), \end{array}\right.$$

where $k>0$. For $k=0$, choose $f_x^0=f_y^0=c_x^0=c_y^0=0$ and $\upsilon _x^0=\upsilon _y^0=\omega _x^0=\omega _y^0=0$.

According to the relaxation algorithm (Jia et al. 2011), we may define,

26 $$\begin{aligned}\left\{ \begin{array}{rcl} f_x^k&=&cut(\nabla _x u^k+f^{k-1}_x,1/\alpha ),\quad f_y^k=cut(\nabla _y u^k+f^{k-1}_y,1/\alpha ), \\ c_x^k&=&cut(\Delta _x u^k+c^{k-1}_x,1/\beta ), \quad c_y^k=cut(\Delta _y u^k+v^{k-1}_y,1/\beta ). \end{array}\right. \end{aligned}$$

So, we have

27 $$u^{k+1}=(1-t)u^k+t\left[ z^k-b^k-\frac{\alpha }{\mu }(1-g)(\nabla ^T_x f^k_x+\nabla ^T_y f^k_y)-\frac{\beta }{\mu }g(\Delta ^T_x c^k_x+\Delta ^T_y f^k_y)\right] ,$$

where $\nabla _x^T$, $\nabla _y^T$, $\Delta _x^T$ and $\Delta _y^T$ are respectively the adjoint operators of $\nabla _x$, $\nabla _y$, $\Delta _x$ and $\Delta _y$. $\nabla _x^T$ and $\nabla _y^T$ have the following discrete forms,

28 $$\begin{aligned} \nabla _x^T u_{i,j}= & \left\{ \begin{array}{ll} -u_{2,j} & \quad if\quad i=1, \\ u_{i,j}-u_{i+1,j} &\quad if\quad 1<i<M, \\ u_{M,j} & \quad if\quad i=M \end{array}\right. \end{aligned}$$

29 $$\begin{aligned} \nabla _y^T u_{i,j}=&\left\{ \begin{array}{ll} -u_{i,2}&\quad if\quad j=1, \\ u_{i,j}-u_{i,j+1} &\quad if\quad 1<j<N, \\ u_{i,N} &\quad if\quad j=N \end{array}\right. \end{aligned}$$

Definitely, $$\Delta _x^T=\Delta _x$$ and $$\Delta _y^T=\Delta _y$$.

#### Solve the second subproblem in Eq. (16)

For the second subproblem in Eq. (16), we derive the Euler–Lagrange equation with respect to *z*, which is as follows,

30 $$(\lambda -\mu \Delta )z=\lambda u_0-\mu \Delta (u^k+b^k).$$

This is a linear equation, so additional operator split (AOS) iteration and Gauss–Seidel (GS) iteration can be used to solve Eq. (30). We use AOS iteration to solve this equation.

In summary, the proposed algorithm for image denoising can be described as follows, 
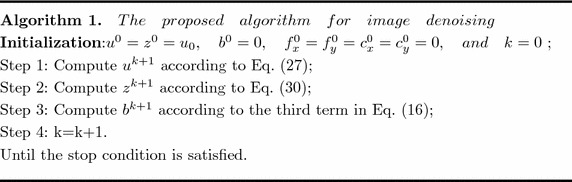


## Experiments

**Fig. 2:**
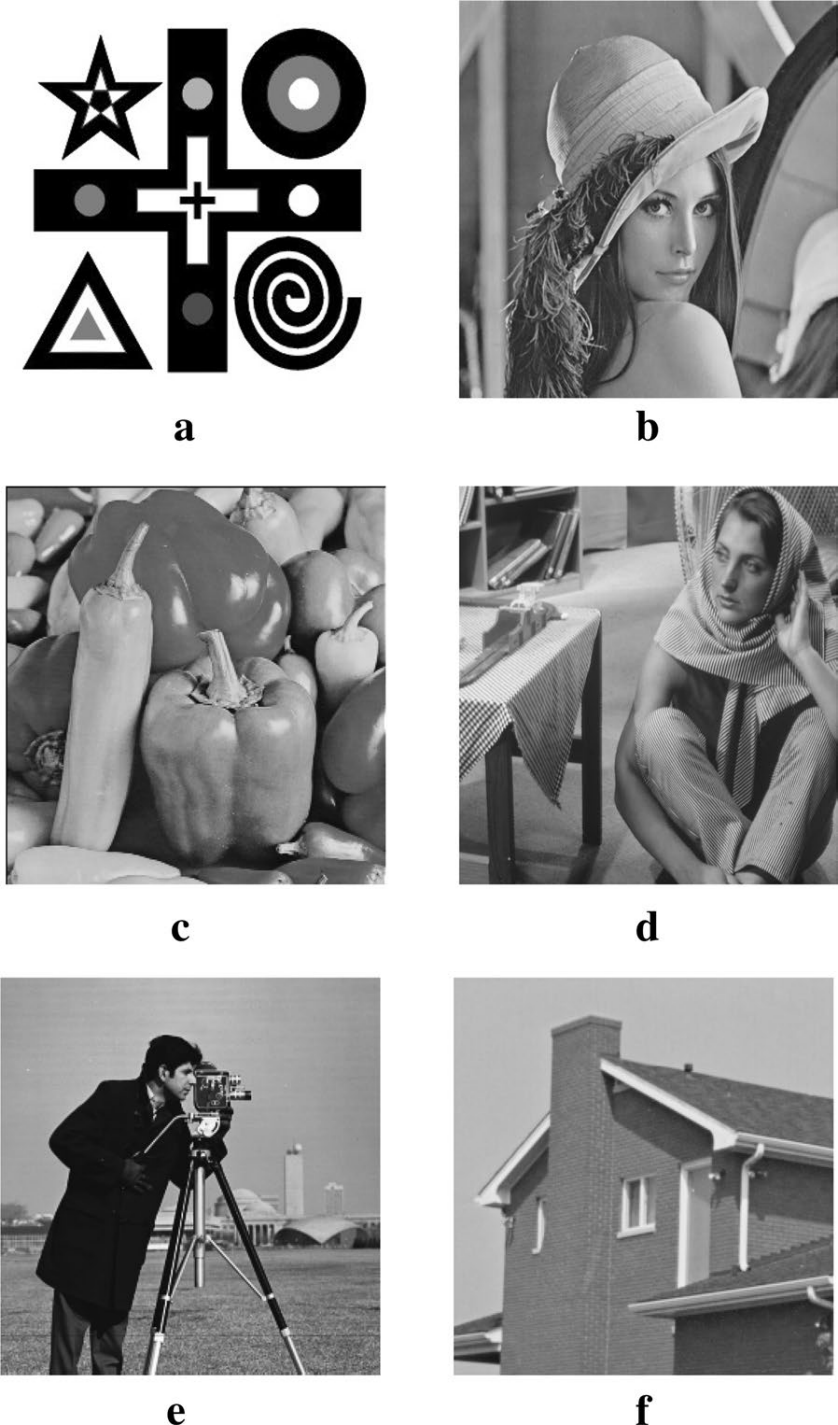
Original test images. **a** Manmade image, **b** lena image, **c** peppers image, **d** barbara image, **e** cameraman image, **f** house image

In this section, we experimentally compare our proposed model with the state-of-the-art models. All experiments are performed under Matlab R2009a on a PC with an Intel CPU of 1.7 GHz and 4 GB memory. Six grayscale images viz. “Manmade”, “Lena”, “Peppers”, “Barbara”, “Cameraman”, and “House” are selected as testing examples for both qualitative and quantitative evaluations. The original test images are shown in Fig. 2. The performances of all methods are compared quantitatively by using the peak signal to noise ratio (PSNR), structural similarity index measure (SSIM) (Wang et al. 2004), multi-scale structural similarity index (MS-SSIM) (Wang et al. 2003), and feature-similarity index (FSIM) (Zhang et al. 2011). In addition, we also compare the computing time and iterations of six models. PSNR is defined as follows,

31 $${\textit{PSNR}}=10\times log_{10}\left( \frac{255^2}{\textit{MSE}}\right) \ (db),$$

with

32 $${\textit{MSE}}(u,{\bar{u}})=\frac{1}{M\times N}\sum \limits _i\sum \limits _j (u_{i,j}-{\bar{u}}_{i,j})^2,$$

where *u* and ${\bar{u}}$ are respectively the recovered image and the original image. Generally, the larger the value of the PSNR, the better the performance. However, PSNR is inconsistent with human visual judgments. SSIM, MS-SSIM, and FSIM are close to the human vision system, so we also use them to assess the noise removal quality. SSIM is defined by,

33 $${\textit{SSIM}}(u,{\bar{u}})=\frac{(2\mu _u\mu _{{\bar{u}}}+c_1)(2\sigma _{u{\bar{u}}}+c_2)}{(\mu ^2_u+\mu ^2_{{\bar{u}}}+c_1)(\sigma ^2_u+\sigma ^2_{{\bar{u}}}+c_2)},$$

where $\mu _u$ and $\sigma _u^2$ are the mean and variance of *u*, respectively, $\sigma _{u{\bar{u}}}$ is the covariance of *u* and ${\bar{u}}$, and $c_1$ and $c_2$ are two constants to avoid instability. MS-SSIM is defined by,

34 $${\textit{MS-SSIM}}(u,{\bar{u}})=[l_M(u,{\bar{u}})]^{\alpha _M}\prod _{i=1}^M[c_i(u,{\bar{u}})]^{\beta _i}[s_i(u,{\bar{u}})]^{\gamma _i},$$

where the luminance distortion $l_i(u,{\bar{u}})$, the contrast distortion $c_i(u,{\bar{u}})$ and the structure distortion $s_i(u,{\bar{u}})$ at scale *i* between images *u* and ${\bar{u}}$ are defined as follows,

35 $$\begin{aligned} \left\{ \begin{array}{lll} l_i(u,{\bar{u}})&=&\frac{2\mu _u\mu _{{\bar{u}}}+c_1}{\mu _u^2+\mu _{{\bar{u}}}^2+c_1};\\ c_i(u,{\bar{u}})&=&\frac{2\sigma _u\sigma _{{\bar{u}}}+c_2}{\sigma _u^2+\sigma _{{\bar{u}}}^2+c_2};\\ s_i(u,{\bar{u}})&=&\frac{2\sigma _{u{\bar{u}}}^2+c_3}{\sigma _u^2\sigma _{{\bar{u}}}^2+c_3}, \end{array}\right. \end{aligned}$$

where $\mu _u$ and $\mu _{{\bar{u}}}$ represent the mean intensity of *u* and ${\bar{u}}$ at scale *i*; $\sigma _u$ (resp. $\sigma _{{\bar{u}}}$) is the standard deviation of *u* (and ${\bar{u}}$) at scale *i*, and $\sigma _{u{\bar{u}}}$ is the covariance between *u* and ${\bar{u}}$ at scale *i*. $c_1$, $c_2$ and $c_3$ are three small constants to avoid instability. In this paper, the values of the exponents $\alpha _M$, $\beta _i$ and $\gamma _i$ are set as the same as those in Wang et al. (2003). FSIM is defined by,

36 $${\textit{FSIM}}=\frac{\sum _{x\in \Omega }S_L(x)PC_m(x)}{\sum _{x\in \Omega }PC_m(x)}$$

where $S_L(x)$ at each location *x* is the similarity measure, which is defined as product of the similarity function $S_{PC}(x)$ on Phase Congruency (PC) and similarity function $S_{G}(x)$ on Gradient Magnitude (GM). $S_{PC}(x)$ and $S_{G}(x)$ are defined as follows,

37 $$\begin{aligned} \left\{ \begin{array}{lll} S_{PC}(x)&=&\frac{2PC_u(x)\cdot PC_{{{\bar{u}}}}(x)+T_1}{PC_u^2(x)+PC_{{{\bar{u}}}}^2(x)+T_1} \\ S_{G}(x)&=&\frac{2G_u(x)\cdot G_{{{\bar{u}}}}(x)+T_2}{G_u^2(x)+G_{{{\bar{u}}}}^2(x)+T_2} \end{array}\right. \end{aligned}$$

where $PC_u$ and $PC_{{{\bar{u}}}}$ denote the PC maps extracted from *u* and ${{\bar{u}}}$, respectively, and $G_u$ and $G_{{{\bar{u}}}}$ denote the GM maps extracted from *u* and ${{\bar{u}}}$, respectively; $T_1$ and $T_2$ are two small positive constants to avoid instability.

The termination condition for all experiments is defined as follows,

38 $$\frac{\Vert u^{n+1}-u^n\Vert ^2_2}{\Vert u^{n+1}\Vert ^2_2}\le \varepsilon,$$

where $u^n$ and $u^{n+1}$ are respectively denoising results at *nth* and $(n+1)th$ iteration, and $\varepsilon$ is a given positive number. We set $\varepsilon =10^{-3}$ in the experiments.

**Fig. 3:**
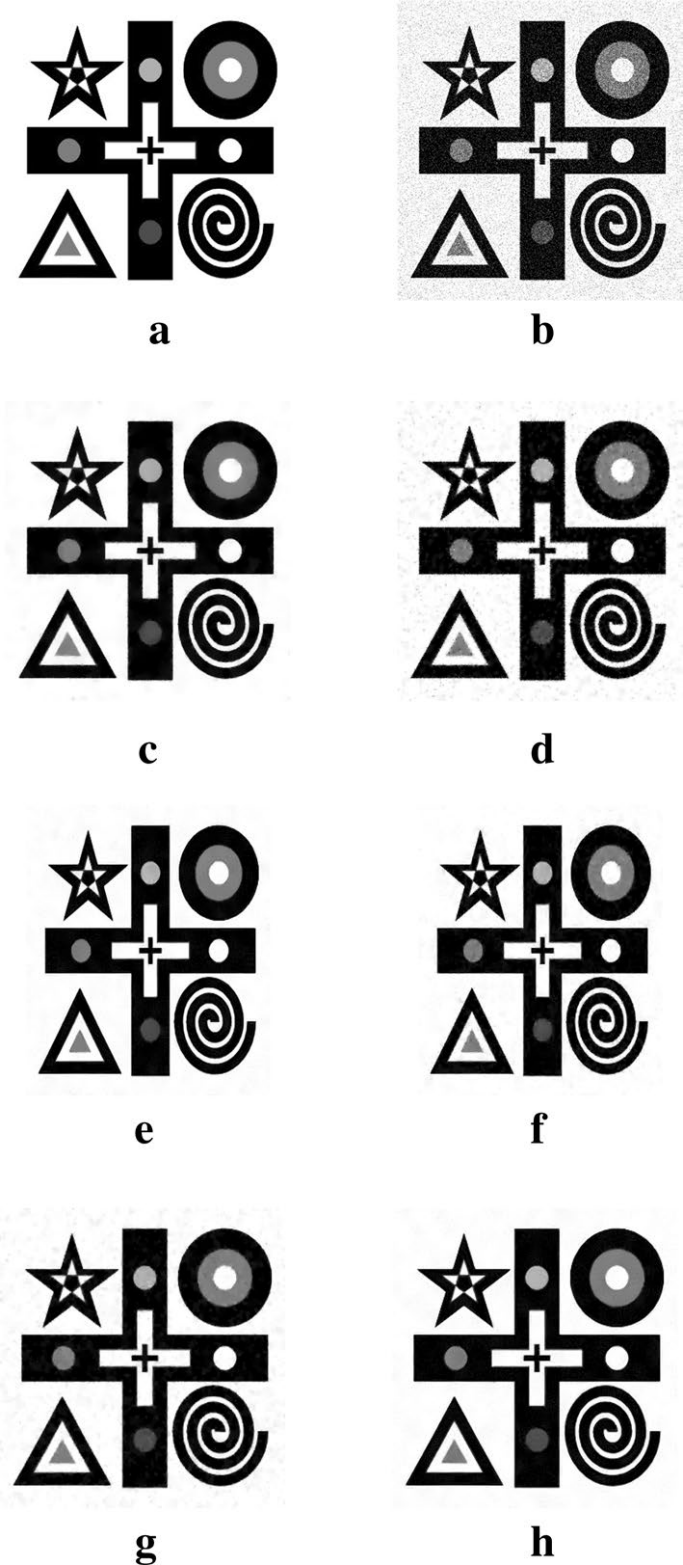
Results of the synthetic image by the six models. **a** Original image, **b** noisy image, **c** result by TV model, **d** result by LLT model, **e** result by NLM model, **f** result by BLS-GSM model, **g** result by hybrid model, **h** result by our model

Figure 3 shows the results for the 8-bit gray-scale synthetic image with size $320\times 320$ pixels, which is corrupted by zero-mean Gaussian white noise with $\sigma =30$. In our experiments, we use the trial-and-error method for determining the optimal parameters. We set $\mu =0.3$, $\alpha =\beta =0.15$, $t=0.2$, ${\mathcal {K}}=0.005$, and $\rho =1$ in our algorithm, while all parameter values in TV model Rudin et al. (1992), LLT model Lysaker et al. (2003), non-local means (NLM) model Buades et al. (2005), BLS-GSM Portilla et al. (2003), and hybrid model Liu (2015) are chosen manually by trial-and-error method to ensure the best results. Figure 3a, b are the original and noisy images, respectively. Figure 3c–h show that the denoising results of TV model, LLT model, NLM model, BLS-GSM, hybrid model, and our proposed model, respectively. Figure 3c indicates that there exists the staircase effects in the recovered images by TV model. Although LLT model has the advantage on relieving the staircase effect, the edges are blurred and there are serious speckles in the recovered image. The computational efficiency of NLM model is very low. There exits the edges blurring in the result of BLS-GSM from Fig. 3f. We also find that there still exits a little stair-case effect by hybrid model from Fig. 3g. Our proposed model relieves the staircasing effects and avoids the edges blurring. Table 1 shows the PSNR, SSIM, MS-SSIM, and FSIM values corresponding to Fig. 3. From Table 1 and Fig. 3, it is shown that our proposed model produces the best result. At the same time, the proposed method takes less computational time than LLT model, NLM model and hybrid model, but the computational time of our proposed method is slightly inferior to that of TV model and BLS-GSM.

**Table 1:** Performance comparison of the recovered results with different methods in Fig. 2

Method	PSNR	SSIM	MS-SSIM	FSIM	Iterations	Time(s)
TV model	31.97	0.91	0.97	0.94	246	11.20
LLT model	30.62	0.86	0.95	0.75	216	87.21
NLM model	32.15	0.89	0.97	0.89	1	275.42
BLS-GSM	31.94	0.92	0.96	0.91	1	4.68
Hybrid model	32.67	0.92	0.96	0.90	197	93.72
Proposed model	33.49	0.93	0.97	0.93	147	81.72

**Fig. 4:**
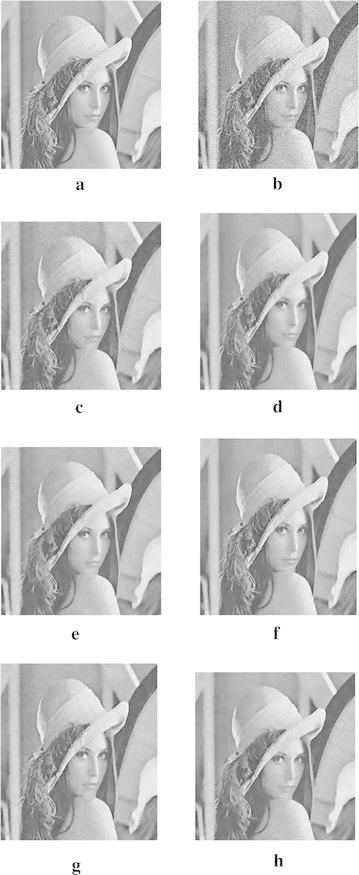
Results of *Lena* image by the six models. **a** Original image, **b** noisy image, **c** result by TV model, **d** result by LLT model, **e** result by NLM model, **f** result by BLS-GSM model, **g** result by hybrid model, **h** result by our model

**Fig. 5:**
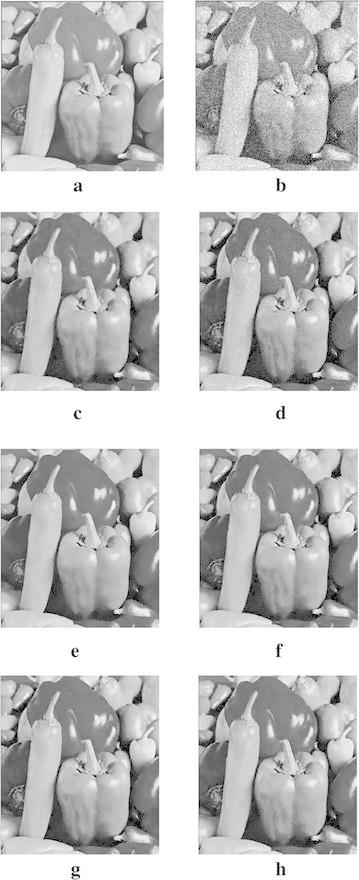
Results of *Peppers* image by the six models. **a** Original image, **b** noisy image, **c** result by TV model, **d** result by LLT model, **e** result by NLM model, **f** result by BLS-GSM model, **g** result by hybrid model, **h** result by our model

**Fig. 6:**
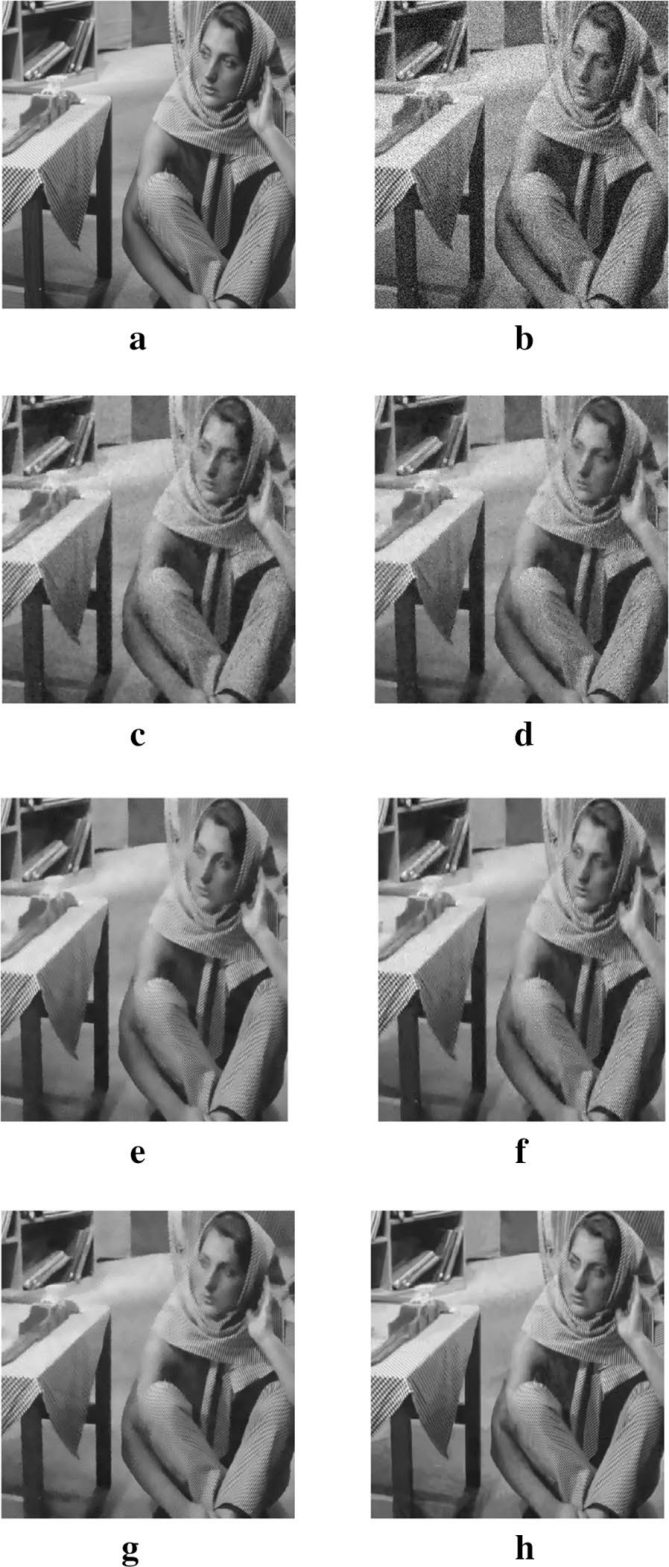
Results of *Barbara* image by the six models. **a** Original image, **b** noisy image, **c** result by TV model, **d** result by LLT model, **e** result by NLM model, **f** result by BLS-GSM model, **g** result by hybrid model, **h** result by our model

**Fig. 7:**
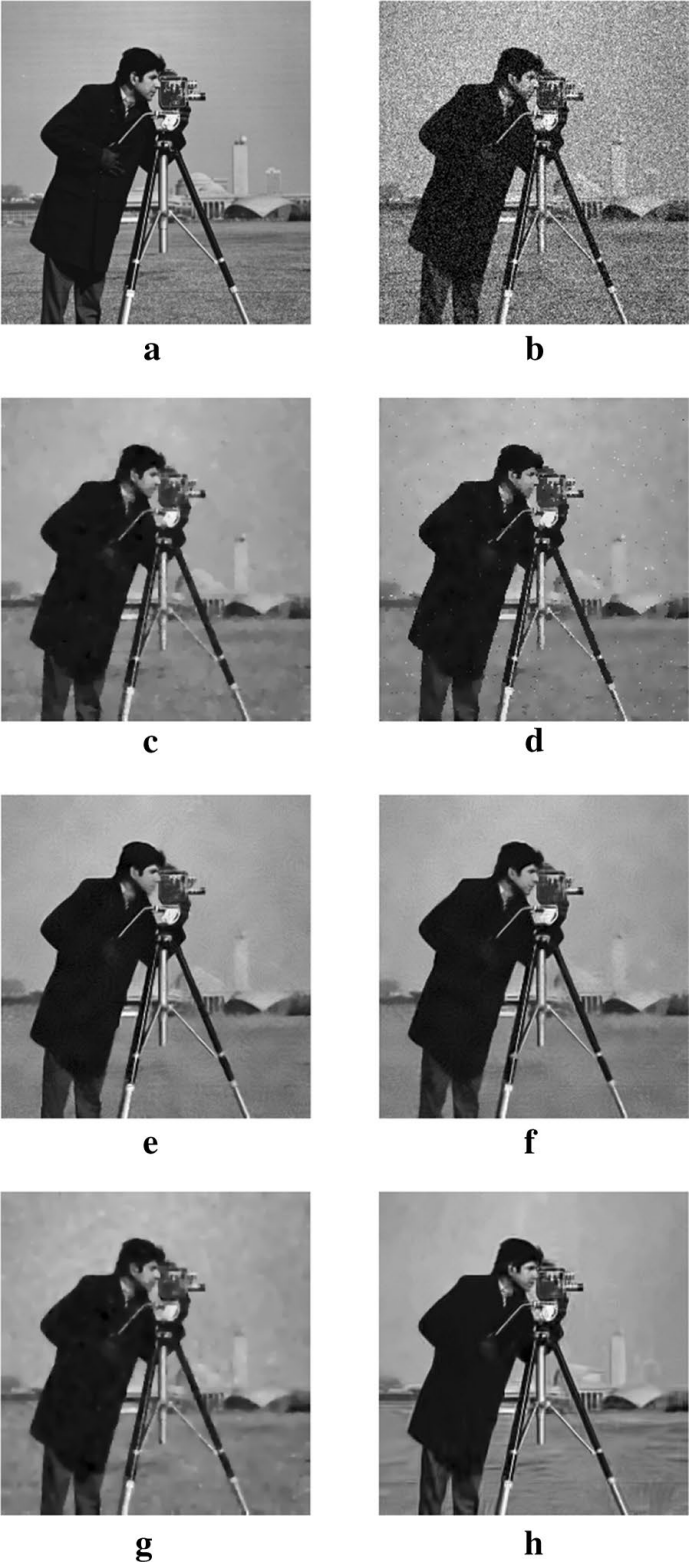
Results of *Cameraman* image by the six models. **a** Original image, **b** noisy image, **c** result by TV model, **d** result by LLT model, **e** result by NLM model, **f** result by BLS-GSM model, **g** result by hybrid model, **h** result by our model

**Fig. 8:**
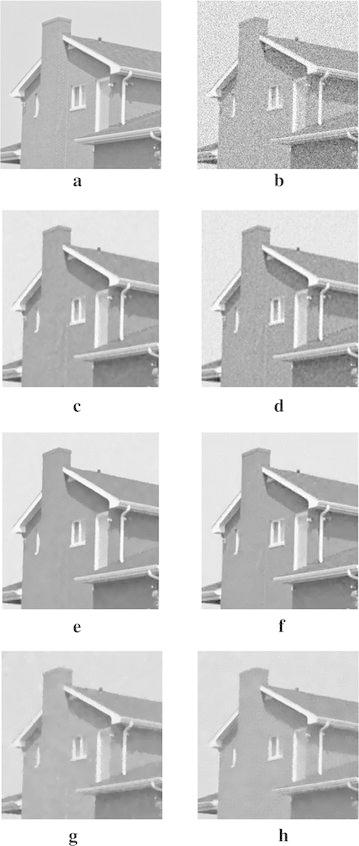
Results of *House* image by the six models. **a** Original image, **b** noisy image, **c** result by TV model, **d** result by LLT model, **e** result by NLM model, **f** result by BLS-GSM model, **g** result by hybrid model, **h** result by our model

**Fig. 9:**
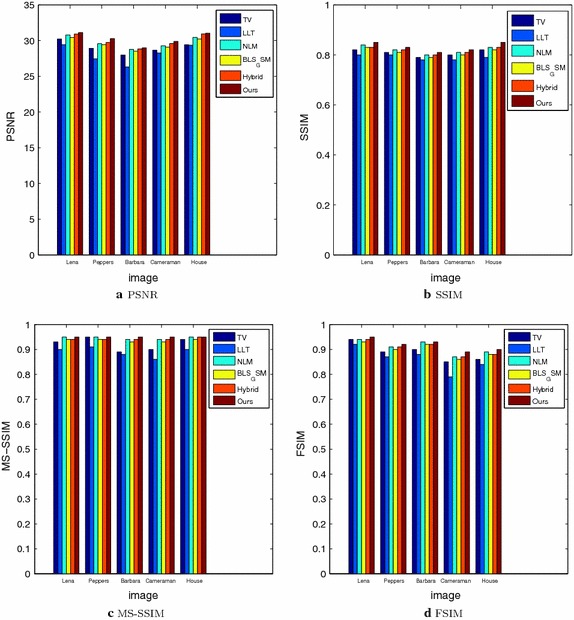
Comparison results in **a** PSNR, **b** SSIM, **c** MS-SSIM and **d** FSIM for Gaussian noise with $\sigma =30$

**Fig. 10:**
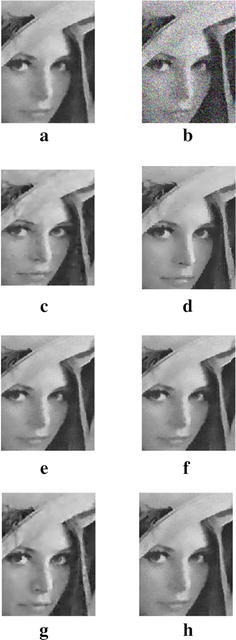
The enlarged detail regions cropped from Fig. 3. **a** Original image, **b** noisy image, **c** result by TV model, **d** result by LLT model, **e** result by NLM model, **f** result by BLS-GSM model, **g** result by hybrid model, **h** result by our model

In order to demonstrate that our model can also work well for natural images, the next experiments are conducted for different images corrupted by zero-mean Gaussian white noise with $\sigma =30$. The experimental results are shown in Figs. 4, 5, 6, 7 and 8, where the experimental results of TV model, LLT model, NLM model, BLS-GSM, hybrid model, and our proposed model are illustrated, respectively. The figures show that our model produces the visually most appealing results among the six models. The quantitative PSNR, SSIM, MS-SSIM, and FSIM values are presented in Fig. 9, which depicts that the performance of our proposed model are better than those of TV model, LLT model, NLM model, BLS-GSM, and hybrid model. In order to verify the better performance of our proposed method, Fig. 10 shows the enlarged regions cropped from Fig. 4.

We also use six images corrupted with different levels of Gaussian noise to examine the performance of the proposed model and the alternative models. Tables 2, 3, 4 and 5 give the PSNR, SSIM, MS-SSIM, and FSIM values obtained by the proposed model and the alternative models, respectively. From Tables 2, 3, 4 and 5, it can be observed that our model is greater than or equal to the other five models in PSNR, SSIM, MS-SSIM, and FSIM for the same standard deviation, which demonstrate that our model provides the best noise removal performance at different noise level.

**Table 2:** Comparison results in PSNR(dB) for different levels of Gaussian noise

Method	Lena	Peppers	Barbara	Cameraman	House
$$\sigma =5$$					
Noise	35.76	34.18	33.15	33.97	35.17
TV model	37.46	36.86	35.96	35.95	37.12
LLT model	36.92	35.94	35.67	35.67	36.34
NLM model	37.41	36.48	36.72	36.76	37.81
BLS-GSM	37.16	36.37	36.18	36.63	37.53
Hybrid model	37.07	36.19	36.11	36.07	37.25
Proposed model	37.44	36.52	36.82	36.81	37.89
$$\sigma =10$$					
Noise	27.72	28.18	28.15	28.12	28.12
TV model	33.72	33.19	30.07	32.39	34.27
LLT model	32.61	31.98	30.12	31.87	32.14
NLM model	34.79	33.35	33.81	33.41	35.41
BLS-GSM	34.64	33.25	33.57	33.26	35.32
Hybrid model	34.56	33.13	33.41	33.22	35.25
Proposed model	35.07	33.86	34.15	34.27	35.64
$$\sigma =20$$					
Noise	22.17	22.16	22.15	22.20	22.18
TV model	31.51	30.54	28.78	29.47	31.21
LLT model	30.67	29.37	27.79	29.32	30.29
NLM model	31.69	31.02	29.92	30.82	31.82
BLS-GSM	31.54	30.76	29.64	30.59	31.54
Hybrid model	31.45	30.71	29.19	30.38	31.43
Proposed model	31.97	31.17	30.32	30.98	32.29
$$\sigma =40$$					
Noise	16.11	16.09	16.12	16.08	16.14
TV model	29.22	27.95	26.93	27.62	28.85
LLT model	28.46	26.07	25.92	27.19	27.95
NLM model	29.57	28.19	27.89	28.59	29.42
BLS-GSM	29.36	28.13	27.67	28.35	29.17
Hybrid model	29.49	28.12	27.86	28.47	29.23
Proposed model	30.13	28.94	28.42	29.15	30.27
$$\sigma =50$$					
Noise	14.17	14.23	14.21	14.16	14.19
TV model	27.51	26.05	24.77	25.89	27.73
LLT model	26.89	25.07	23.91	25.11	26.84
NLM model	27.95	26.21	25.69	26.32	27.06
BLS-GSM	27.67	26.03	25.43	26.13	26.74
Hybrid model	27.76	26.14	25.17	26.27	26.97
Proposed model	28.21	26.97	25.88	26.62	27.37

**Table 3:** Comparison results in SSIM for different levels of Gaussian noise

Method	Lena	Peppers	Barbara	Cameraman	House
$$\sigma =5$$					
Noise	0.85	0.85	0.89	0.84	0.80
TV model	0.93	0.91	0.92	0.93	0.97
LLT model	0.91	0.89	0.93	0.92	0.94
NLM model	0.94	0.92	0.96	0.94	0.97
BLS-GSM	0.94	0.92	0.96	0.96	0.98
Hybrid model	0.94	0.93	0.96	0.96	0.98
Proposed model	0.94	0.93	0.97	0.97	0.97
$$\sigma =10$$					
Noise	0.61	0.61	0.71	0.63	0.53
TV model	0.89	0.88	0.87	0.86	0.90
LLT model	0.87	0.86	0.85	0.86	0.89
NLM model	0.90	0.89	0.90	0.90	0.92
BLS-GSM	0.91	0.88	0.91	0.92	0.92
Hybrid model	0.91	0.88	0.90	0.90	0.91
Proposed model	0.91	0.89	0.91	0.91	0.90
$$\sigma =20$$					
Noise	0.34	0.43	0.48	0.41	0.35
TV model	0.86	0.85	0.82	0.83	0.84
LLT model	0.84	0.83	0.80	0.82	0.83
NLM model	0.87	0.86	0.83	0.85	0.85
BLS-GSM	0.86	0.84	0.83	0.84	0.87
Hybrid model	0.87	0.85	0.82	0.83	0.85
Proposed model	0.89	0.87	0.84	0.86	0.87
$$\sigma =40$$					
Noise	0.15	0.21	0.26	0.22	0.16
TV model	0.79	0.77	0.75	0.77	0.80
LLT model	0.75	0.74	0.73	0.74	0.76
NLM model	0.82	0.80	0.76	0.76	0.82
BLS-GSM	0.79	0.77	0.74	0.76	0.83
Hybrid model	0.81	0.79	0.76	0.78	0.81
Proposed model	0.82	0.79	0.75	0.79	0.83
$$\sigma =50$$					
Noise	0.11	0.17	0.15	0.18	0.13
TV model	0.73	0.72	0.69	0.71	0.73
LLT model	0.70	0.69	0.65	0.69	0.70
NLM model	0.73	0.74	0.71	0.70	0.75
BLS-GSM	0.74	0.73	0.69	0.72	0.75
Hybrid model	0.73	0.73	0.70	0.71	0.74
Proposed model	0.74	0.74	0.72	0.73	0.76

**Table 4:** Comparison results in MS-SSIM for different levels of Gaussian noise

Method	Lena	Peppers	Barbara	Cameraman	House
$$\sigma =5$$					
Noise	0.97	0.98	0.98	0.97	0.97
TV model	0.99	0.99	0.99	0.98	0.99
LLT model	0.96	0.99	0.95	0.96	0.97
NLM model	0.98	0.99	0.99	0.98	0.98
BLS-GSM	0.99	0.99	0.98	0.98	0.98
Hybrid model	0.98	0.98	0.98	0.97	0.97
Proposed model	0.99	0.99	0.99	0.99	0.99
$$\sigma =10$$					
Noise	0.93	0.95	0.95	0.93	0.92
TV model	0.97	0.98	0.97	0.97	0.97
LLT model	0.96	0.97	0.95	0.96	0.96
NLM model	0.98	0.98	0.98	0.98	0.98
BLS-GSM	0.98	0.98	0.97	0.98	0.98
Hybrid model	0.97	0.97	0.98	0.99	0.98
Proposed model	0.98	0.98	0.98	0.99	0.98
$$\sigma =20$$					
Noise	0.83	0.89	0.88	0.83	0.82
TV model	0.95	0.97	0.92	0.93	0.96
LLT model	0.94	0.95	0.92	0.91	0.93
NLM model	0.97	0.97	0.96	0.96	0.97
BLS-GSM	0.96	0.96	0.96	0.95	0.97
Hybrid model	0.96	0.95	0.96	0.95	0.96
Proposed model	0.97	0.96	0.97	0.96	0.96
$$\sigma =40$$					
Noise	0.66	0.76	0.73	0.68	0.65
TV model	0.91	0.93	0.87	0.89	0.92
LLT model	0.88	0.89	0.85	0.82	0.85
NLM model	0.93	0.94	0.92	0.93	0.94
BLS-GSM	0.93	0.93	0.91	0.93	0.94
Hybrid model	0.92	0.93	0.90	0.92	0.93
Proposed model	0.93	0.94	0.92	0.94	0.94
$$\sigma =50$$					
Noise	0.60	0.70	0.67	0.63	0.60
TV model	0.89	0.91	0.85	0.89	0.90
LLT model	0.84	0.86	0.82	0.80	0.82
NLM model	0.91	0.92	0.89	0.91	0.92
BLS-GSM	0.91	0.92	0.88	0.90	0.92
Hybrid model	0.90	0.91	0.88	0.89	0.91
Proposed model	0.92	0.92	0.90	0.91	0.92

**Table 5:** Comparison results in FSIM for different levels of Gaussian noise

Method	Lena	Peppers	Barbara	Cameraman	House
$$\sigma =5$$					
Noise	0.99	0.96	0.99	0.95	0.95
TV model	0.99	0.97	0.99	0.97	0.97
LLT model	0.99	0.97	0.99	0.97	0.97
NLM model	0.99	0.98	0.99	0.98	0.97
BLS-GSM	0.99	0.97	0.99	0.98	0.97
Hybrid model	0.99	0.97	0.98	0.97	0.96
Proposed model	0.99	0.99	0.99	0.98	0.98
$$\sigma =10$$					
Noise	0.95	0.87	0.96	0.86	0.86
TV model	0.98	0.95	0.96	0.93	0.93
LLT model	0.97	0.94	0.97	0.93	0.93
NLM model	0.98	0.97	0.98	0.95	0.95
BLS-GSM	0.98	0.96	0.97	0.95	0.94
Hybrid model	0.97	0.95	0.96	0.94	0.94
Proposed model	0.98	0.96	0.97	0.96	0.95
$$\sigma =20$$					
Noise	0.86	0.73	0.88	0.72	0.71
TV model	0.95	0.91	0.92	0.88	0.88
LLT model	0.94	0.90	0.92	0.86	0.88
NLM model	0.96	0.94	0.94	0.91	0.92
BLS-GSM	0.95	0.93	0.94	0.90	0.91
Hybrid model	0.94	0.92	0.93	0.89	0.91
Proposed model	0.96	0.93	0.94	0.91	0.92
$$\sigma =40$$					
Noise	0.72	0.54	0.74	0.56	0.53
TV model	0.93	0.86	0.89	0.81	0.84
LLT model	0.91	0.81	0.86	0.76	0.82
NLM model	0.93	0.89	0.92	0.85	0.88
BLS-GSM	0.92	0.88	0.92	0.84	0.87
Hybrid model	0.92	0.87	0.91	0.84	0.87
Proposed model	0.94	0.89	0.92	0.85	0.88
$$\sigma =50$$					
Noise	0.65	0.48	0.67	0.51	0.48
TV model	0.89	0.83	0.87	0.79	0.82
LLT model	0.86	0.78	0.84	0.73	0.79
NLM model	0.91	0.88	0.90	0.83	0.85
BLS-GSM	0.90	0.87	0.89	0.82	0.85
Hybrid model	0.89	0.86	0.88	0.81	0.84
Proposed model	0.91	0.88	0.90	0.82	0.85

## Conclusions

To eliminate the so-called staircase effect in total variation filter and avoid the edges blurring for fourth-order PDE filter, we propose an adaptive anisotropic diffusion model for image denoising, which is composed of a hybrid regularization term combining a total variation filter and a fourth-order filter and the fidelity term using the $H^{-1}$-norm. We also develop an efficient algorithm to solve our proposed model. Numerical experiments show that our proposed model has the highest PSNR, SSIM, MS-SSIM, and FSIM values among the six methods, and can preserve important structures, such as edges and corners.
